# Vitamin-A deficiency and its determinants among preschool children: a community based cross-sectional study in Ethiopia

**DOI:** 10.1186/s13104-016-2134-z

**Published:** 2016-06-24

**Authors:** Amare Tariku, Abel Fekadu, Ayanaw Tsega Ferede, Solomon Mekonnen Abebe, Akilew Awoke Adane

**Affiliations:** Department of Human Nutrition, Institute of Public Health, College of Medicine and Health Sciences, The University of Gondar, Gondar, Ethiopia; Department of Epidemiology and Biostatistics, Institute of Public Health, College of Medicine and Health Sciences, The University of Gondar, Gondar, Ethiopia; Department of Optometry, School of Medicine, College of Medicine and Health Sciences, The University of Gondar, Gondar, Ethiopia

**Keywords:** Xerophthalmia, Determinants, Preschool children, Ethiopia

## Abstract

**Background:**

Vitamin A deficiency is the leading cause of preventable visual impairments in children. It is also an underlying cause for nearly one-fourth of global child mortality associated with measles, diarrhea, and malaria. The limited literature available in Ethiopia shows severe public health significance of vitamin-A deficiency. Hence the aim of the current study was to assess the prevalence and factors determining vitamin-A deficiency among preschool children in Dembia District, northwest Ethiopia.

**Methods:**

A community-based cross-sectional study was conducted among preschool children of Dembia District from January to February, 2015. A multi-stage sampling, followed by a systematic sampling technique was employed to select study participants. A structured interviewer-administered questionnaire was used to collect data. Using a binary logistic regression model, multivariable analysis was fitted to identify the associated factors of vitamin-A deficiency. The adjusted odds ratio (AOR) with a 95 % confidence interval was computed to assess the strength of the association, and variables with a p value of <0.05 in multivariable analysis were considered as statistically significant.

**Results:**

Six hundred eighty-one preschool children were included in the study, giving a response rate of 96.5 %. The overall prevalence of xerophthalmia was 8.6 %. The result of the multivariable analysis revealed that nonattendance at the antenatal care clinic [AOR 2.65,95 % CI (1.39,5.07)], being male [AOR 1.81, 95 % CI (1.01,3.24)], and in the age group of 49–59 months [AOR 3.00, 95 % CI (1.49,6.02)] were significantly associated with vitamin-A deficiency.

**Conclusions:**

Vitamin-A deficiency is a severe public health problem in the study area. Further strengthening antenatal care utilization and giving emphasis to preschool children will help to mitigate vitamin-A deficiency in the study area.

## Background

Vitamin A is an essential nutrient needed in smaller amounts for normal visual and immune functions, the maintenance of epithelial cellular integrity, growth, and development [1, 2]. Due to their increased nutrient demand and the severity of the potential health consequences associated with vitamin-A deficiency (VAD), preschool children and pregnant women are considered as the most at-risk segments of the community [1]. According to the recent World Health Organization (WHO) estimate, VAD has a moderate and severe public health significance in 45 and 122 countries in the world, respectively. About one-third (33.3 %) of the world’s preschool children are found with sub-clinical VAD, and 0.9 % with night blindness. The highest burden of VAD occurs in Africa and southeast Asia [1]. Africa alone contributes more than one-third of the global burden of childhood xerophthalmia [3], according to which about 44.4 % (56.4 million) and 2 % of the preschool children are affected by sub-clinical VAD and night blindness, respectively.

The consequence of VAD is magnified by poverty and the higher prevalence of infectious diseases [4] and it is an underlying cause for nearly one-fourth of global child mortality from measles, diarrhea, and malaria [5]. This mortality risk worsens among children born in Sub-Saharan African countries, which face 16.5 and 1.8 times higher probability of dying before the age of 5 years compared to children born in developed regions and Southern Asia, respectively [6]. VAD along with measles is the major cause of preventable visual impairment in children [7]. Though VAD is a multi-casual disorder, episodes of severe disease [8–10], poor dietary intake of protein and vitamin-A rich food [10], lack of vitamin-A supplementation, poor immunization status, poor maternal awareness about vitamin-A and high parity [8, 9], poor maternal education, socioeconomic status and sanitary practice, male sex, and nutritional stunting [11, 12] were some of the factors significantly associated with it.

The findings of the limited research done in Ethiopia reveal that, VAD is one of the major public health problems [3, 13–15], because more than one-third (37. 6 %) of the children under 5 years are suffering from sub-clinical VAD, 4.3–7.3 % from night blindness, and 2.2 % from Bitot’s Spots [14]. In Ethiopia and other African countries, poverty, sub-optimal nutrition, insanitary living conditions, and poor health care access exacerbate the risk of developing multiple micronutrient deficiencies [16–18]. Accordingly, only half of the young children grow with optimal breastfeeding. Twenty-six percent of the young children consume vitamin-A rich foods, while only 4 % receive food from at least four food groups [19].

It has been confirmed that improving the vitamin A status of deficient children significantly reduces the risk of mortality from measles by 50 %, from diarrhea by 40 %, and overall mortality by 25–35 % [20]. Therefore, the elimination of VAD is considered a key element for improving the survival, well-being, growth, and development of children. Thus, research showing the burden and determinants of VAD is of paramount importance. However, such studies are scarce in Ethiopia, particularly in the northwestern part of the country. Therefore, this study aimed to assess the prevalence and determinants of VAD among preschool children.

## Methods

### Study setting

The study was conducted in Dembia District, northwest Ethiopia. The district has 45 kebeles *(smallest administrative units in Ethiopia),* of which 40 are rural. It has a total population of 315,903 with 260.1/km^2^ population density. Preschoolers comprise 5.7 % (18,006) of the total population [21]. There are 10 health centers and 40 health posts in the district. Surrounded by the great Lake Tana, the district is a well-known malaria endemic area. The residents are by and large surplus producing farmers cultivating mainly cereals, legumes, and spices [22]. According to the 2015 District Health Office report, the overall immunization coverage is 81.5 % while it is 77.1 % for measles. Furthermore, the coverage for Rota Virus-1 and Rota Virus-2 immunization is 83.1 and 77.1 %, respectively, and incredibly, it is 98 % for vitamin-A supplementation.

### Study design and participants

A community-based cross-sectional study was conducted from January to February, 2015. All preschool children who lived in the district for at least 6 months were included in the study.

### Sample size and sampling procedure

The minimum sample size was determined using the single population proportion formula with the following assumptions: 5.8 % expected prevalence of xerophthalmia [15], 95 % confidence level, and 2.5 % margin of error (d). Finally, a minimum sample size of 706 was obtained after anticipating a 10 % non-response rate and the adjusting design effect of two. A multi-stage sampling followed by a systematic sampling technique was employed to reach the study subjects. Initially, nine representative kebeles in the district (one urban and eight rural) were selected using the lottery method. The total number of eligible preschool children living in the kebeles were obtained from the local administration and used to calculate the sampling fraction (k). After a proportional allocation to each kebele, the systematic sampling technique was employed to reach the study subjects. In households with more than one eligible study subject, lottery was used to select only one child. When mother–child pairs were not available at the time of data collection, two repeated visits were made. Otherwise, the adjacent house was considered, though rarely.

### Data collection instruments and procedure

Data were collected through a face to face interview by using a pretested and structured questionnaire. The questionnaire consisted of socio-demographic and economic characteristics, health, and dietary pattern related information. To maintain consistency, the questionnaire was first translated from English to Amharic, the native language of the study area, and was retranslated to English by professional translators. Two experienced public health experts and 12 trained data collectors (2 clinical optometrists and 10 clinical nurses) were recruited for supervision and data collection, respectively. The investigators coordinated the overall activities of data collection. The tool was piloted on 36 preschool children outside the study area. During the pre-test, the acceptability and applicability of the procedures and tools were evaluated. Household wealth index was computed using a composite indicator for urban and rural residents by considering properties like, livestock ownership, selected household assets, size of agricultural land, and the quantity of crop production. Principal component analysis (PCA) was performed to categorize the household wealth index into lowest, middle, and highest.

Height was measured using the seca vertical height scale (German, Serial No. 0123) standing upright in the middle of the board. The child’s head, shoulders, buttocks, knees, and heels touch the vertical board. Nutrition related data were transferred to the ENA/SMART software version 2012 and hight-for-Age Z-scores (HAZ) was calculated using the WHO Multicenter Growth Reference Standard. Children were classified as undernourished if their z score was less than −2SD; otherwise, they were well-nourished (≥−2 z score). Accordingly, children were considered as stunted if their z-score of HAZ was below −2SD, respectively.

### Assessment of dietary diversity and quasi-food frequency

Determination of the dietary diversity score (DDS) of a child was started by asking the mother to list all foods consumed by the child in the previous 24 h preceding the survey. In case of mixed dish, mothers were asked to list the ingredients of the food items. Then reported food items were classified into seven food groups, as starchy staples (grains, roots, and tubers); legumes, nuts and seeds; vitamin-A rich fruits and vegetables; other fruits and vegetables; egg; dairy products (milk, yoghurt, and cheese); and flesh foods (meat, fish, poultry, and organ meats) [23]. Considering four food groups as the minimum acceptable dietary diversity [24], a child with a DDS of less than four was classified as having poor dietary diversity; otherwise, they were considered to have good dietary diversity.

The seven-day quasi food frequency, modified food group frequency [2], was measured as the number of days the child consumed any of the vitamin-A rich foods in the last 7 days preceding the survey. Primarily, vitamin-A rich foods available in the study area were classified into plant-based (dark green leafy vegetables, other vitamin-A rich vegetables, and yellow orange fruits) and animal-based food groups (organ meat, eggs, and milk) [25]. Mothers were asked to report for how many days their children consumed any of the vitamin-A rich foods in the last 7 days. A score ranging from 1 to 7 was given depending on the number of days the child ate any of the above listed vitamin-A rich foods; otherwise, the score ‘0’ was given if the child didn’t eat any vitamin-A rich food. Hence, the above food groups constituted the six food groups which accounted for the maximum score of seven for each; the total score for the summary measure of quasi-food frequency was 42.

### Clinical assessment of vitamin-A deficiency

A detailed ophthalmic examination was carried out by clinical optometrists with a strict adherence to standard methods and procedures [26]. An ophthalmoscope, a pentorch, and magnifiers were used to identify the clinical signs of vitamin-A deficiency, such as Bitot’s spot, conjunctival xerosis, corneal xerosis, corneal ulceration, and corneal scar. But, history of night blindness was confirmed by asking mothers using the local word for night blindness *(dafint)*. Information on whether a child faced any difficulty while playing or in identifying objects in dim light, especially at sun set, was gathered.

### Data analysis

Data were entered into EPI INFO version 3.5.3 and analyzed using the Statistical Package for Social Sciences (SPSS) version 20. Descriptive statistics, including frequencies and proportions were used to summarize the study variables. A binary logistic regression model was used to investigate factors associated with VAD. Variables with a p values of <0.2 in the bivariable analysis were entered in the multivariable analysis to control the possible effect of confounders. The adjusted odds ratio (AOR) with a 95 % confidence interval was estimated to assess the strength of association, and a p value of <0.05 was used to declare the statistical significance in the multivariable analysis. Furthermore the fitness of the model was checked using the Hosmer and lemeshow goodness of fit-test, and it was found as 0.68.

## Results

### Socio-demographic characteristics

Six hundred eighty-one children were included in the study. The mean age (±SD) of the children was 41.58 months (±11.27), and slightly more than half (53.6 %) of them were male. Almost all (93.1 %) of the participants were living in the rural *kebeles* of Dembia District. In this community, nearly one-third (30 %) of the households (HHDs) had at least seven family members. The majority (95.4 %) of the mothers were housewives, uneducated (77.1 %), and gave their first birth before the age of 20 (63.1 %). Most (84.4 %) of the parents obtained food for household consumption from their own farm (own production) (Table 1).

**Table 1:** Socio-demographic characteristics of study participants in Dembia District, northwest Ethiopia, 2015

Variables	Frequency	Percent
Child age in month		
24–36	288	42.3
37–48	233	34.2
49–59	160	23.5
Child sex		
Male	365	53.6
Female	316	46.4
Residence		
Urban	47	6.9
Rural	634	93.1
Marital status		
Single	25	3.7
Married	613	90
Others^a^	43	6.3
Religion		
Orthodox	674	99
Others^b^	7	1
Ethnicity		
Amhara	673	98.8
Others^f^	8	1.2
Household size		
≤4	226	33.2
5–6	251	36.8
≥7	204	30.0
Number of children ever born		
≤2	195	28.6
3–5	355	52.1
≥6	131	19.2
Birth order		
1st	128	18.8
2nd–4th	362	53.2
≥5th	191	28
Maternal education		
Uneducated	525	77.1
Primary	58	8.5
Secondary and above	98	14.4
Maternal employment		
Housewife	650	95.4
Others^c^	31	4.6
Maternal age		
15–34	495	72.7
35–48	186	27.3
Mothers age at first birth		
≤19	430	63.1
20–39	251	36.9
Paternal education		
Uneducated	410	60.2
Educated	271	39.8
Paternal employment		
Farmer	620	91
Merchant	32	4.7
Others^d^	29	4.3
Wealth status		
Poor	226	33.2
Middle	228	33.5
High	227	33.3
Supported by PSNP		
Yes	41	6.0
No	640	94.0
Model household graduate		
Graduated	285	41.9
Not graduated	396	58.1
Main source of family food		
Own production	575	84.4
Other^e^	106	15.6
Vegetable production		
Yes	108	15.9
No	573	84.1
Fruit production		
Yes	33	4.8
No	648	95.2

^a^Divorced, widowed and separated

^b^Muslim and protestant

^c^Merchant, government employ and student

^d^Government employ and daily laborer

^e^Purchasing and family assistant

^f^Oromo and Tigre

### Health and nutrition related characteristics

**Fig. 1:**
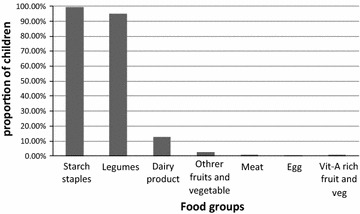
Proportion of preschool children who consumed food groups in the previous 24-h preceding the survey, Dembia District, northwest Ethiopia, 2015

The majority (85.8 %) of the mothers had at least one antenatal care (ANC) visit for the index child, but around one third (29.9 %) gave birth at heath facilities. A substantial number (92.4 %) of the children took vitamin-A supplementation in the last 6 months. Most (82.5 %) of the children had a dietary diversity score of below 4. Only a few children ate meat (0.6 %), eggs (0.3 %), and vitamin-A rich fruits and vegetables (0.6 %) in the previous 24-h (Fig. 1). Furthermore, about 36 % of them ate any of vitamin-A rich food in the last 7 days preceding the survey. Nearly three-fourths (70.3 %) of the mothers initiated breast feeding in a timely fashion, within an hour of delivery, and a significant proportion of the mothers (65.9 %) initiated complementary feeding at the 6 month (Table 2).

**Table 2:** Health and nutrition related characteristics of study participants in Dembia District, northwest Ethiopia, 2015

Variables	Frequency	Percent
ANC visit		
Yes	584	85.8
No	97	14.2
Place of delivery		
Home	480	70.5
Health facility	201	29.5
Initiation of breastfeeding		
Early initiation	479	70.3
Late initiation	202	29.7
Prelacteal feeding		
Yes	344	50.5
No	337	49.5
Complementary food initiation		
Timely	449	65.9
Early	33	4.8
Late	199	29.2
Dietary diversity		
<4	562	82.5
≥4	119	17.5
Quasi food frequency		
Zero	438	64.3
1–3	137	20.1
≥4	106	15.6
Vitamin-A supplementation		
Yes	629	92.4
No	52	7.6
Immunization status		
Partially immunized	121	17.8
Fully immunized	560	82.2
Any morbidity in the last 2 week		
Yes	108	15.9
No	573	84.1
Stunting		
Yes	313	46
No	368	54

### Prevalence of vitamin-A deficiency

**Fig. 2:**
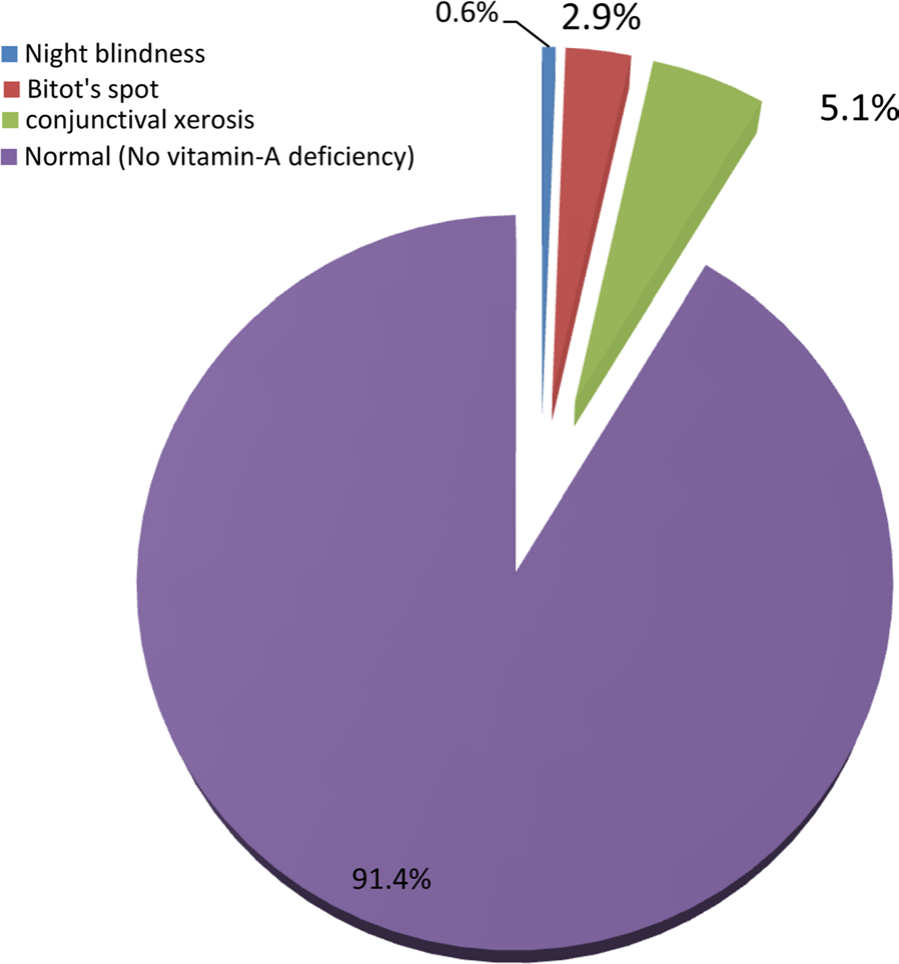
Prevalence of vitamin-A deficiency among preschool children in Dembia District, northwest Ethiopia, 2015

The overall prevalence of xerophthalmia was 8.6 % [95 % CI 6.6,11 %]. In the following diagram each specific type was summarized (Fig. 2).

### Factors associated with vitamin-A deficiency

In both the bivariable and multivariable analyses, sex and age of the child and the ANC follow up were significantly associated with VAD. Accordingly, being a male child increases the odds of developing VAD 1.81 times [AOR 1.81,95 % CI 1.01,3.24]. Likewise, increased odds of VAD were noted among children aged 49-59 months [AOR 3.00, 95 % CI 1.49,6.02] and whose mothers had no ANC visit [AOR 2.65, 95 % CI 1.39,5.07] (Table 3).

**Table 3:** Factors associated with vitamin-A deficiency among preschool children in Dembia District, northwest Ethiopia, 2015

Variables	Vitamin-A deficiency		COR (95 % CI)	AOR (95 % CI)
	Yes# (%)	No# (%)		
Child sex				
Male	40 (11 %)	325 (89 %)	1.92 (1.09,3.39)	1.81 (1.01,3.24)
Female	19 (6 %)	297 (94 %)	1	1
Wealth status				
Poor	17 (7.5 %)	209 (92.5 %)	0.66 (0.35,1.25)	
Middle	17 (7.5 %)	211 (92.5 %)	0.65 (0.34,1.24)	
High	25 (11 %)	202 (89 %)	1	
Child age in month				
24–36	16 (5.6 %)	272 (94.4 %)	1	1
37–48	20 (8.6 %)	213 (91.4 %)	1.59 (0.81,3.16)	1.57 (0.78,3.17)
49–59	23 (14.4 %)	137 (85.6 %)	2.85 (1.46,5.58)	3.00 (1.49,6.02)
Place of delivery				
Home	46 (9.6 %)	434 (90.4 %)	1	
Health facility	13 (6.5 %)	188 (93.5 %)	0.65 (0.34,1.24)	
Model household				
Yes	20 (7 %)	265 (93 %)	0.69 (0.39,1.21)	
No	39 (9.8 %)	257 (90.2 %)	1	
Quasi food frequency				
Zero	44 (10 %)	394 (90 %)	1.58 (0.69,3.61)	
1–3	8 (5.8 %)	129 (94.2 %)	0.88 (0.31,2.5)	
≥4	7 (6.6 %)	99 (93.4 %)	1	
Antenatal care				
Yes	42 (7.2 %)	542 (92.8 %)	1	1
No	17 (17.5 %)	80 (82.5 %)	2.74 (1.49,5.05)	2.65 (1.39,5.07)
Vegetable production				
Yes	13 (12 %)	95 (88 %)	1.57 (0.82,3.02)	
No	46 (8 %)	527 (92 %)	1	
Main source of family food				
Own production	54 (9.4 %)	521 (90.6 %)	1	
Other	5 (4.7 %)	101 (95.3 %)	0.48 (0.19, 1.22)	
Number of children ever born				
≤2	21 (10.8 %)	174 (89.2 %)	2.57 (0.97,6.41)	
3–5	32 (9.0 %)	323 (91 %)	2.06 (0.84,5.06)	
≥6	6 (4.6 %)	125 (95.4 %)	1	

## Discussion

In this study, in spite of intensive nutritional interventions, the prevalence of Xerophthalmia still remains unacceptably high, five times higher than the WHO cut-off point for public health significance (1.56 %) [27]. This might be related to the lower rate of maternal literacy in the study area (22.9 %). Maternal literacy is a strong predictor of good childhood nutritional outcome, mainly through creating a fertile ground for adoption of scientifically supported child feeding practices [28, 29]. Moreover, poor dietary intake of vitamin-A rich food is an important predictor of VAD [9, 30, 31]. Thus, the higher burden of xerophthalmia in the area could also be attributed to poor dietary intake of vitamin-A rich food. Nearly two-thirds (64.3 %) of the children didn’t eat any of the vitamin-A rich foods in the last 7 days preceding the survey, indicating a higher vulnerability of the children to VAD [26, 27].

This prevalence was also the highest of any study reporting from developing countries, such as Nigeria (1.1 %) [32] and rural India (2.3 %) [12]. This huge discrepancy could be partially explained by the socio-economic and cultural differences of the study areas.

Children aged 49–59 months were more likely to suffer from VAD as compared to children who were between 24 and 36 months of age. Similar findings were reported in Sudan [33] and India [34]. This is probably due to the fact that this age category is the turning point for increased energy and micronutrient requirements compared to younger children, to support their rapid growth and development [35, 36]. Though their physiological state calls for extra nutritional requirement, only few children consumed vitamin-A rich complementary food in the current study area and in the country at large [28]. This extra micronutrient requirement coupled with poor dietary intake might worsen the risk of developing VAD in older children.

The current study also found that male children were at higher risk of developing vitamin-A deficiency. This finding was supported by reports from Israel [11] and India [34]. However a different finding was reported by another study in Ethiopia [37]. This might be related to the slightly higher nutritional requirement of male children for vitamin-A [38]. The other possible explanation could be related to diarrheal diseases, as most male children (89.8 %) experienced fever and diarrhea in this study. According to some reports, fever is mostly associated with the commonest childhood illness of infectious origin, such as diarrhea, malaria, and respiratory tract infection. Febrile illness during childhood could result in decreased food intake and malabsorption of nutrients, such as vitamin-A. It is also confirmed that, the above highly common childhood infectious diseases are significant determinants of VAD [10, 31, 39]. However, the relationship between child sex and risk of VAD needs further investigation.

The third important factor associated with VAD was ANC follow up. Children whose mothers had no ANC follow up were more likely to suffer from VAD. ANC is an ideal entry point for providing nutritional care and counseling [40] and to promote the benefit of institutional delivery [41, 42]. Proper nutritional counseling and support helps to enhance nutritional knowledge and appropriate dietary habit of pregnant mothers [43]. ANC also steps-up the likelihood of receiving the child and maternal postnatal vitamin-A supplementation [44]. This in turn could help to improve maternal vitamin-A reserve for later lactation. Additionally, ANC with a profound effect of institutional delivery helps to promote exclusive and early initiation of breastfeeding [45, 46], commonly advocated public health measures to avert childhood VAD [13, 28].

This study tried to show the burden of vitamin-A deficiency in preschool children, which has not been well investigated before in the study area. The investigators also made a lot of effort to maintain the quality of the data, mainly through a pretest, frequent field supervisions, and training of data collectors. However, the study was not free from limitations. First, since dietary assessments were made through recall (with the longest recall period of 7 days in case of the seven-day quasi-frequency), there might have been a risk of recall bias. Secondly, the study didn’t consider the measurement of the serum retinol level and estimation of the portion size of food intake. Thirdly, the study was not free from the pitfalls of a cross-sectional study design.

## Conclusions

Vitamin-A deficiency has severe public health significance in the study area. Strengthening the utilization of antenatal care and giving special emphasis to preschool children helps to mitigate vitamin-A deficiency.

## Abbreviations

WHO: World Health Organization
AOR: adjusted odds ratio
CI: confidence interval
COR: crude odds ratio
ANC: antenatal care (ANC)
VAD: vitamin-A deficiency
DDS: dietary diversity score
HHDs: households
WHZ: weight-for-hight Z-scores
HAZ: hight-for-age Z-scores
